# Supplementary material for: A novel in silico scaffold-hopping method for drug repositioning in rare and intractable diseases
Source: Sci Rep. 2023 Nov 8;13:19358. doi: 10.1038/s41598-023-46648-1 (PMC10632405; doi:10.1038/s41598-023-46648-1)
Supplement: Supplementary file 1 — Supplementary Information. [file 41598_2023_46648_MOESM1_ESM.pdf]

Supplementary Information

A novel *in silico* scaffold-hopping method for drug repositioning in rare and intractable diseases

Mao Tanabe, Ryuichi Sakate, Jun Nakabayashi, Kyosuke Tsumura, Shino Ohira, Kaoru Iwato, Tomonori Kimura

CONTENTS	Page No
Table S1: The DrugBank indexes of the compounds with the highest AAM similarity among the hits whose known targets were the same as or different from those of the reference.	3
Table S2: The classification of hits and non-hits based on whether or not their pharmacological action type is same as the reference.	4
Table S3: Contingency table for function data of hits and non-hits screened on the basis of testosterone.	5
Figure S1: HPLC results.	6
Figure S2: Chemical structures of aldosterone as a reference, and the hits and non-hits whose known targets are the same as that of the reference (i.e., NR3C2).	7
Figure S3: Chemical structures of testosterone as a reference, and some hits and non-hits whose known targets are the same as that of the reference (i.e., AR).	8
Figure S4: Chemical structures of sildenafil as a reference, and the hits and non-hits whose known targets are the same as that of the reference (i.e., PDE5A).	9
Figure S5: Chemical structures of sunitinib as a reference, and some hits and non-hits whose known targets are the same as that of the reference (e.g., KIT).	10
Figure S6: Chemical structures of celecoxib as a reference, and some hits and non-hits whose known targets are the same as that of the reference (i.e., PTGS2).	11
Figure S7: The hit rate of the compounds targeting the same protein as the reference compound when AI-AAM is not applied or applied.	12
Figure S8: The free energy of compound binding to the target.	13
Figure S9: AAM similarity of selective COX2 inhibitors and non-selective NSAIDs identified with celecoxib as a reference.	14
Figure S10: Hit compounds (reference: BIIB-057) classified on the basis of the biological functions of their known targets.	15

Supplementary Table S1: The DrugBank indexes of the compounds with the highest AAM similarity among the hits whose known targets were the same as or different from those of the reference.

Target	cmpd. (same target)	cmpd. (different target)
NR3C2	DB12221	DB14681
NR3C4	DB01406	DB00990
PDE5	DB06267	DB15110
KIT	DB11973	DB12147
PTGS2	DB11395	–

Supplementary Table S2: The classification of hits and non-hits based on whether or not their pharmacological action type is same as the reference.

**Reference compound**			**The compounds that target the same protein as the reference compound**		
Name	Target	Pharmacological action type	Pharmacological action type	Hits (N)	Non-hits (N)
Aldosterone	NR3C2	**Agonist**	**Agonist**	3	0
			Antagonist	4	4
Testosterone	AR	**Agonist**	**Agonist**	14	0
			Antagonist/ Modulator	4	6*
Sildenafil	PDE5A	Inhibitor	Inhibitor	2	1
Sunitinib	KIT	**Type I inhibitor** **	**Type I inhibitor**	3	1
			Type II inhibitor	1	3
			Unknown	6	6
Celecoxib	PTGS2	**Selective COX-2 inhibitor**	**Selective COX-2 inhibitor**	2	5
			Non-selective NSAIDs	0	10
			CINOD	0	1

*Antagonist: 5, Modulator: 1

**Wang, B et al. (2021)[25], Zhao, Z et al. (2014)[26]

Supplementary Table S3: Contingency table for function data of hits and non-hits screened on the basis of testosterone.

	Agonist	Antagonist	SUM
Hits	14	4	18
Non-hits	0	5	5
SUM	14	9	23

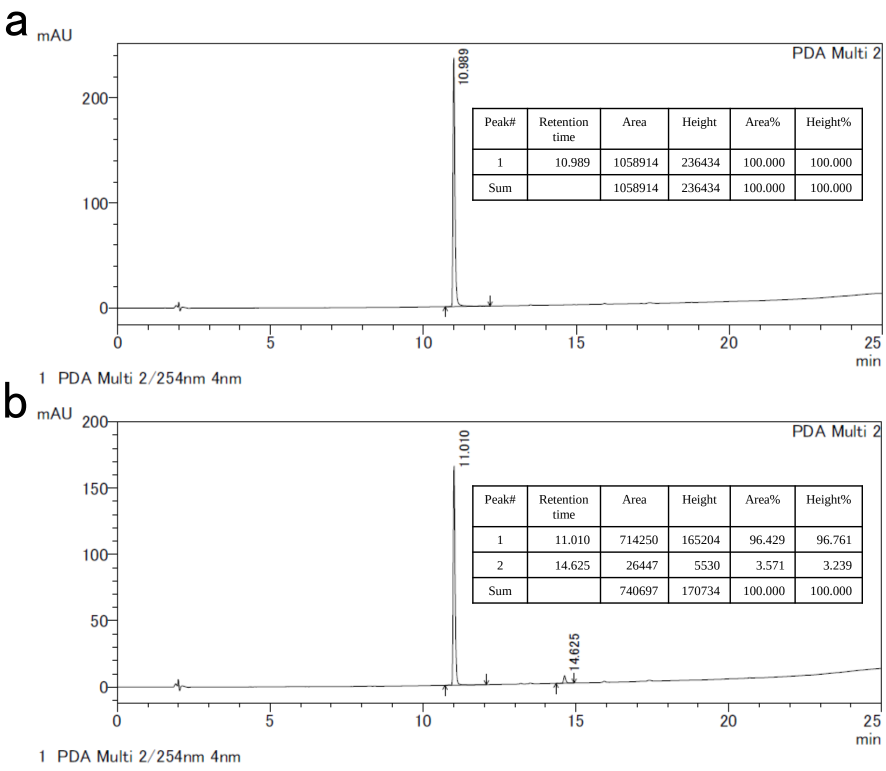

Supplementary Figure S1: HPLC results.

a BIIB-057. **b** XC608.

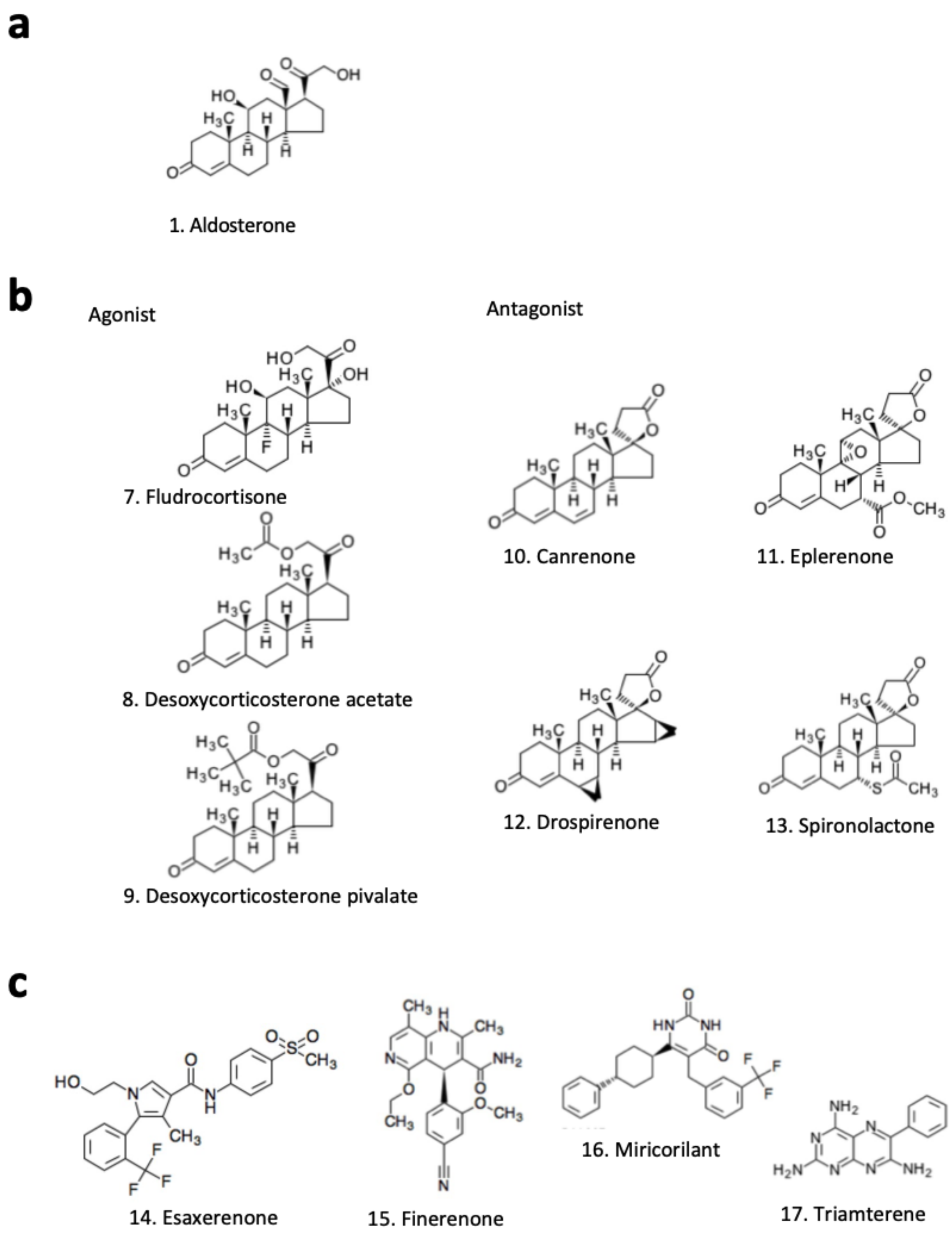

Supplementary Figure S2: Chemical structures of aldosterone as a reference, and the hits and non-hits whose known targets are the same as that of the reference (i.e., NR3C2).

a Aldosterone as a reference. **b** Seven hits. **c** Four non-hits.

a

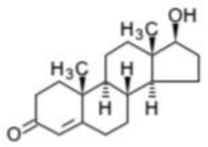

2. Testosterone

b

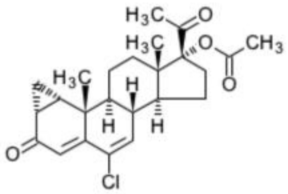

18. Cyproterone acetate

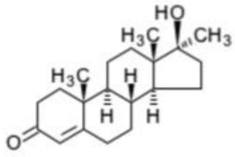

19. Methyltestosterone

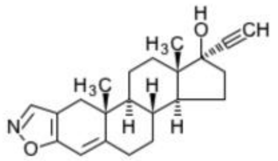

20. Danazol

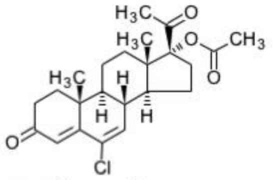

21. Chlormadinone acetate

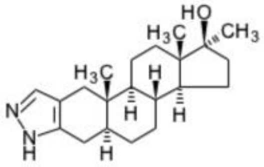

22. Stanozolol

c

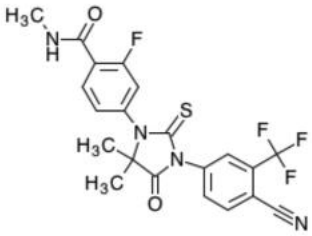

23. Enzalutamide

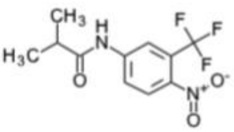

24. Flutamide

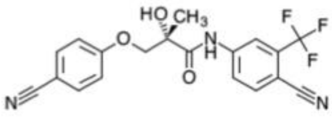

25. Enobosarm

Supplementary Figure S3: Chemical structures of testosterone as a reference, and some hits and non-hits whose known targets are the same as that of the reference (i.e., AR).

a Testosterone as a reference**. b** Five hits. **c** Three non-hits.

a

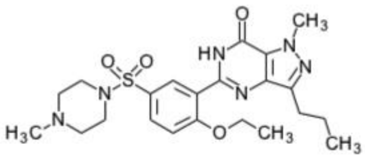

3. Sildenafil

b

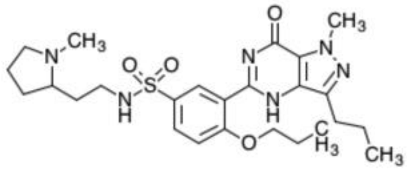

26. Udenafil

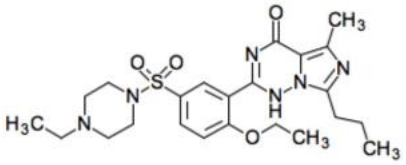

27. Vardenafil

c

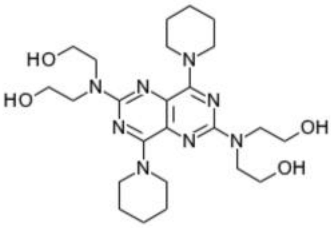

28. Dipyridamole

Supplementary Figure S4: Chemical structures of sildenafil as a reference, and the hits and non-hits whose known targets are the same as that of the reference (i.e., PDE5A).
a Sildenafil as a reference. **b** Two hits. **c** A non-hit.

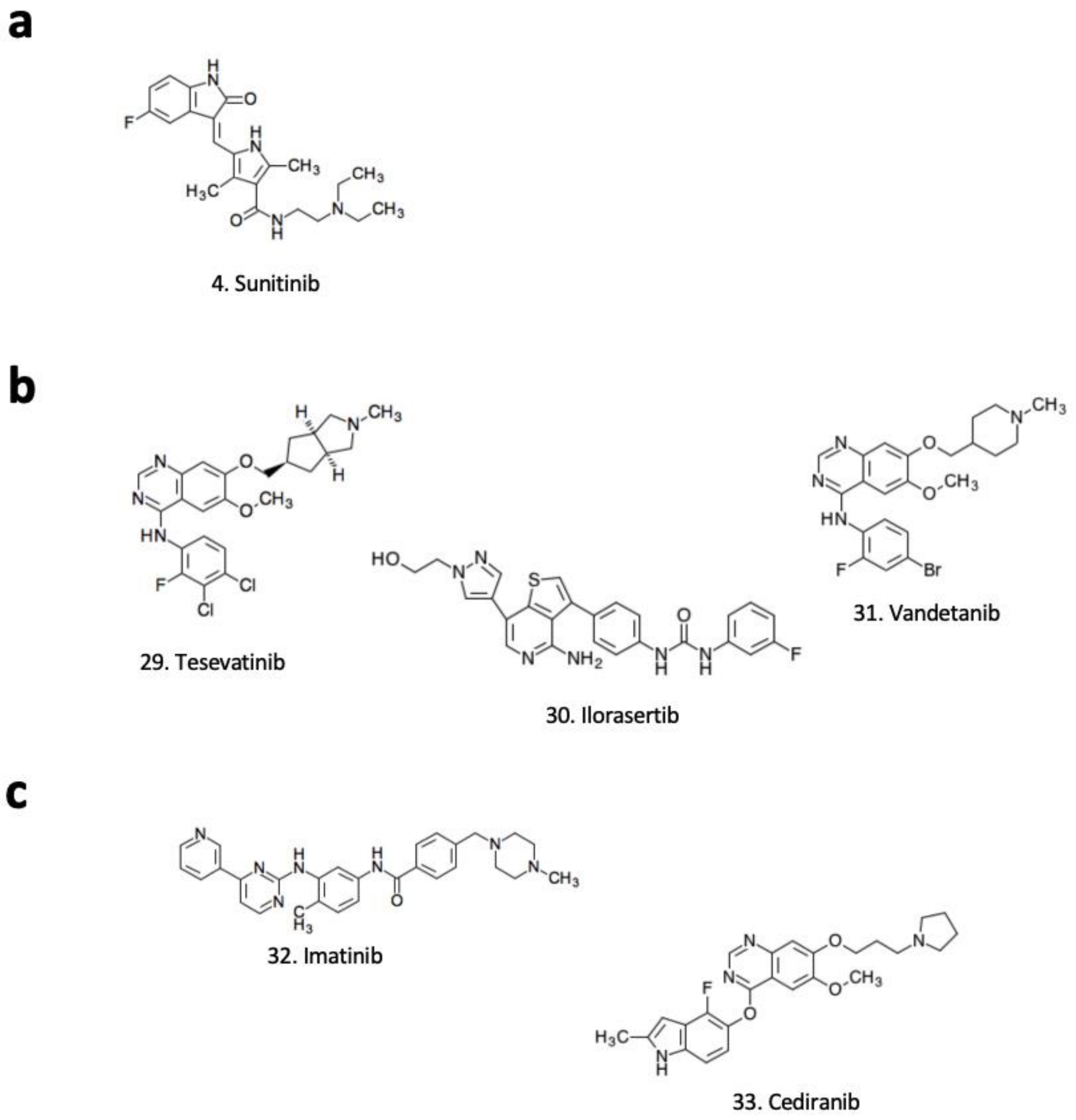

Supplementary Figure S5: Chemical structures of sunitinib as a reference, and some hits and non-hits whose known targets are the same as that of the reference (e.g., KIT).

a Sunitinib as a reference. **b** Three hits. **c** Two non-hits.

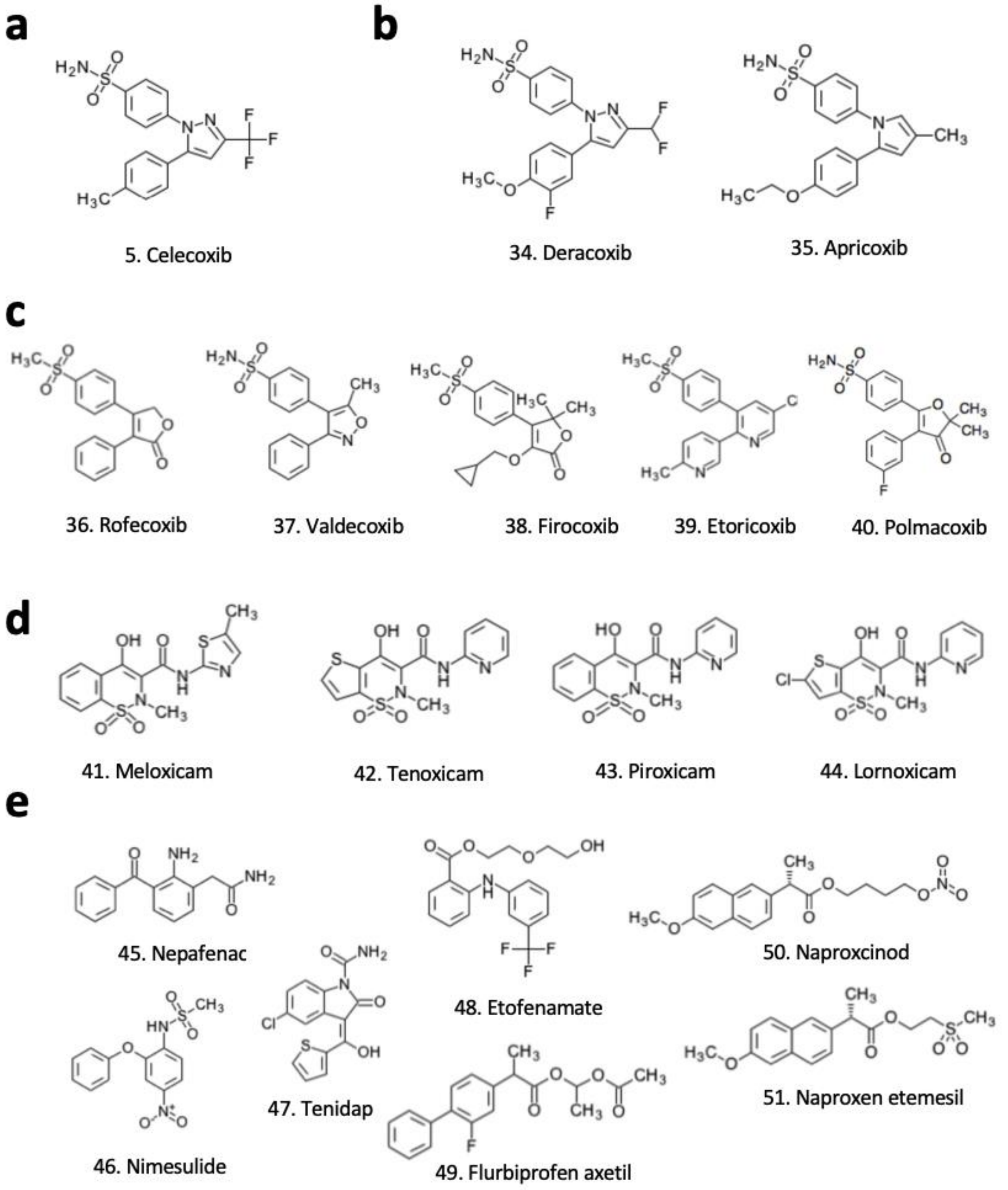

Supplementary Figure S6: Chemical structures of celecoxib as a reference, and some hits and non-hits whose known targets are the same as that of the reference (i.e., PTGS2).

a Celecoxib as a reference. **b** Two hits. **c** Five non-hits known as celecoxib analogues. **d** Four non-hits known as meloxicam analogues. **e** The other non-hits.

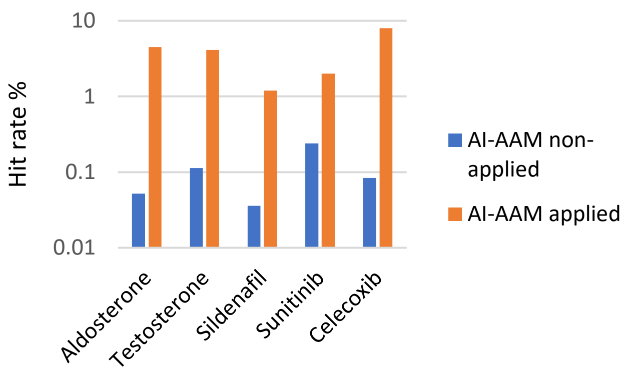

Supplementary Figure S7: The hit rate of the compounds targeting the same protein as the reference compound when AI-AAM is not applied or applied.

The graph uses logarithmic scale on the vertical axis.

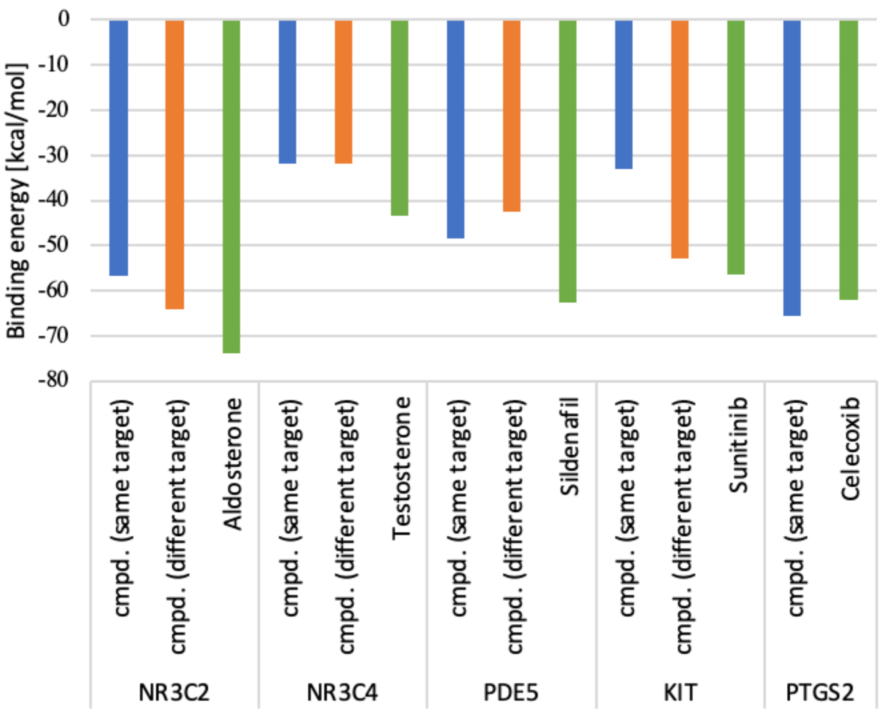

Supplementary Figure S8: The free energy of compound binding to the target.

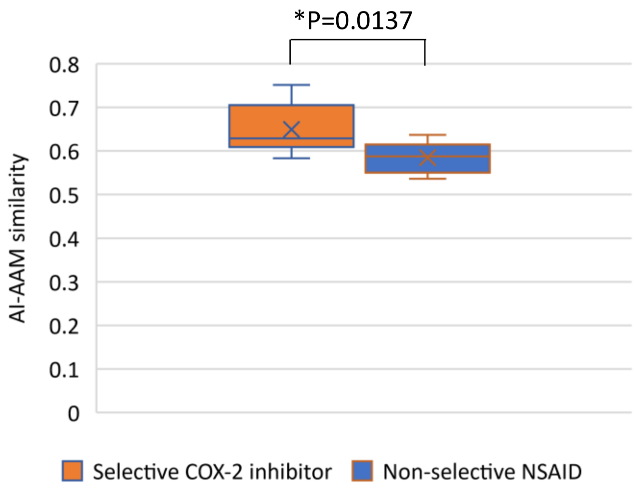

Supplementary Figure S9: AAM similarity of selective COX2 inhibitors and non-selective NSAIDs identified with celecoxib as a reference.

Each box-and-whisker plot shows the five-number summary of a set of data for AI-AAM similarity of selective COX-2 inhibitors (n=7) and non-selective NSAIDs (n=10), respectively. A box is drawn from the first quartile to the third quartile. A vertical line goes through the box at the median. The whiskers go from the ends of the box to the minimum or maximum values. P value was calculated by two-tailed unpaired t-test. The asterisk indicates the statistically significant difference ($p < 0.05$).

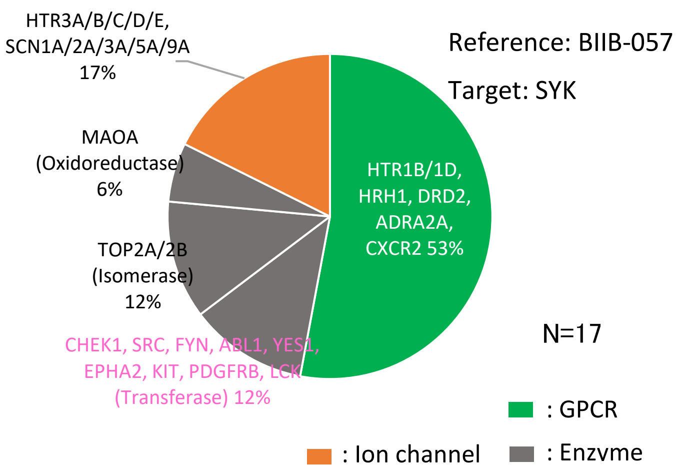

Supplementary Figure S10: Hit compounds (reference: BIIB-057) classified on the basis of the biological functions of their known targets.

Pink letters represent the same targets as the reference compounds.
